# Improving In Vivo Brain Delivery of Monoclonal Antibody Using Novel Cyclic Peptides

**DOI:** 10.3390/pharmaceutics11110568

**Published:** 2019-10-31

**Authors:** Kavisha R. Ulapane, Brian M. Kopec, Teruna J. Siahaan

**Affiliations:** Department of Pharmaceutical Chemistry, The University of Kansas, 2095 Constant Avenue, Lawrence, KS 66047, USA; k581h718@ku.edu (K.R.U.); brian.kopec@ku.edu (B.M.K.)

**Keywords:** blood–brain barrier (BBB), cadherin cyclic peptides, monoclonal antibody (mAb), paracellular pathway, mAb in vivo brain delivery

## Abstract

Many proteins can be used to treat brain diseases; however, the presence of the blood–brain barrier (BBB) creates an obstacle to delivering them into the brain. Previously, various molecules were delivered through the paracellular pathway of the BBB via its modulation, using ADTC5 and HAV6 peptides. This study goal was to design new cyclic peptides with N-to-C terminal cyclization for better plasma stability and modulation of the BBB. Cyclic HAVN1 and HAVN2 peptides were derived from a linear HAV6 peptide. Linear and N-to-C terminal cyclic ADTHAV peptides were designed by combining the sequences of ADTC5 and HAV6. These novel cyclic peptides were used to deliver an IRdye800CW-labeled IgG monoclonal antibody into the brain. Cyclic HAVN1 and HAVN2 peptides deliver IgG into the brain, while the parent linear HAV6 peptide does not. Cyclic and linear ADTHAV and ADTC5 peptides enhanced brain delivery of IgG mAb, in which cyclic ADTHAV peptide was better than linear ADTHAV (*p* = 0.07). Cyclic ADTHAV and ADTC5 influenced the distribution of IgG mAb in other organs while HAV6, HAVN1 and HAVN2 did not. In summary, the novel cyclic peptides are generally better BBB modulators than their linear counterparts for delivering IgG mAb into the brain.

## 1. Introduction

Insufficient delivery of drugs across the blood–brain barrier (BBB) has become the major challenge in diagnosing and treating brain-related diseases [1,2,3]. Many large molecule therapeutics, such as neurotrophic factors, enzymes, and antibodies, are being developed to treat brain disorders such as neurodegenerative diseases (e.g., multiple sclerosis (MS) and Alzheimer’s disease (AD)) and brain tumors (i.e., glioblastoma and medulloblastoma). Unfortunately, the BBB is one of the major hurdles for brain delivery of large therapeutic agents such as proteins. Most large molecules, such as monoclonal antibodies (mAbs) with a molecular weight around 150 kDa, have a low ability to enter the brain because any molecule that crosses the BBB requires appropriate physicochemical properties for passive diffusion or a specific transporter for an active transport mechanism. The BBB is a very selective barrier, main function of which is to regulate the passage of nutrients into the brain and provide brain protection against toxic compounds and pathogens. The transcellular pathway, which many hydrophobic drugs utilize to cross the BBB, is also limited to molecules with physicochemical properties that at least satisfy Lipinski’s rule of five [4]. Even if a molecule satisfies Lipinski’s rules, efflux pumps can still prevent it from crossing the BBB [2]. 

The paracellular pathway, where a drug molecule can pass through intercellular junctions, is also limited to small ions and hydrophilic molecules with hydrodynamic radius <11 Å [2]. This is due to the presence of tight junctions between two opposing cellular membranes. Therefore, large hydrophilic molecules such as peptides and proteins normally cannot passively diffuse through the paracellular pathway. Conversely, a select number of peptides and proteins (e.g., insulin, transferrin) can cross the BBB via the transcellular pathway because they have specific transporter systems (e.g., insulin and transferrin receptors) [3,5,6,7,8,9]. Currently, many neurotrophic factors such as nerve growth factor (NGF) [10] and brain-derived neurotrophic factor (BDNF) [11] have been investigated for treating neurodegenerative diseases such as MS and AD. Unfortunately, as with other proteins, the brain delivery of NGF or BDNF is still very challenging. Therefore, there is an urgent need to develop methods that are safe and effective for improving delivery of proteins and peptides into the brain.

Several attempts have been made to overcome this challenge using various invasive and non-invasive methods. Intracerebral ventricular (ICV) delivery is an invasive injection technique in which drug substances are administered directly into the cerebrospinal fluid in cerebral ventricles. Alternatively, many non-invasive methods have been investigated in vivo with various levels of success using receptor-mediated drug delivery systems, liposomes, microbubble-enhanced diagnostic ultrasound (MEUS), and nanoparticles [12,13,14]. The most successful non-invasive method for delivering drugs to brain tumor patients is osmotic BBB disruption, which utilizes a mannitol hypertonic solution to shrink the vascular endothelial cells and increase the porosity of the paracellular pathway to improve permeation of antitumor drugs from the blood into the brain [15,16,17]. Following in the footsteps of the osmotic BBB method, we designed cadherin peptides (i.e., HAV6 and ADTC5) that modulate cadherin–cadherin interactions in the adherens junctions to increase the porosity of the BBB paracellular pathways, so as to allow molecules to cross from the bloodstream into the brain [1,2]. The proposed mechanism is that cadherin peptides inhibit cadherin-mediated cell–cell adhesion in a specific, dynamic, and equilibrium fashion. Both HAV6 and ADTC5 peptides were derived from the extracellular-1 (EC-1) domain of E-cadherin and, collectively, they have been shown to increase the brain delivery of various molecules, including paracellular markers (i.e., ^14^C-mannitol, 25 kDa IRdye800CW-polyethylene glycols or PEG), anticancer drugs (i.e., ^3^H-daunomycin, Glu-CPT, adenanthin), efflux pump substrates (i.e., rhodamine 800 (R800), ^3^H-daunomycin), magnetic resonance imaging (MRI)-enhancing agents (i.e., gadopentetic acid or Gd-DTPA), peptides (i.e., IRdye800CW-cLABL and cIBR7), and proteins (i.e., 65 kDa galbumin) [18,19,20,21,22,23]. Recently, HAV6 has been shown to significantly improve the delivery of an anticancer drug, adenanthin, into the brains of mice with medulloblastoma brain tumor. Treatments with a combination adenanthin and HAV6 increased survival and suppressed tumor growth compared to treatments with adenanthin alone or placebo [24]. Similarly, oral and brain deliveries of eflornithine in rats were simultaneously increased by HAV6 peptide [25].

**Figure 1:**
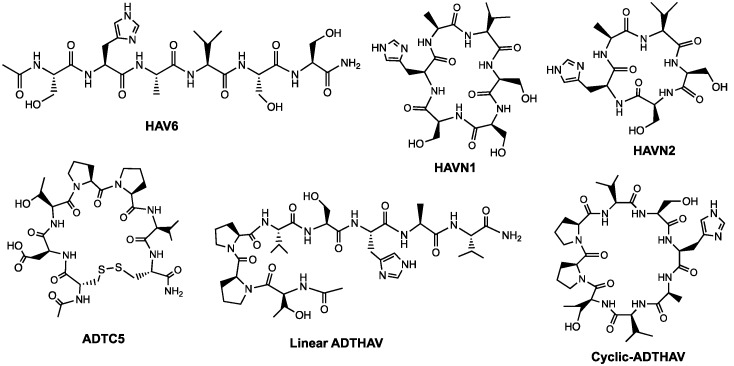
Chemical structures of HAV6, HAVN1, HAVN2, ADTC5, linear ADTHAV, and cyclic ADTHAV peptides.

**Figure 2:**
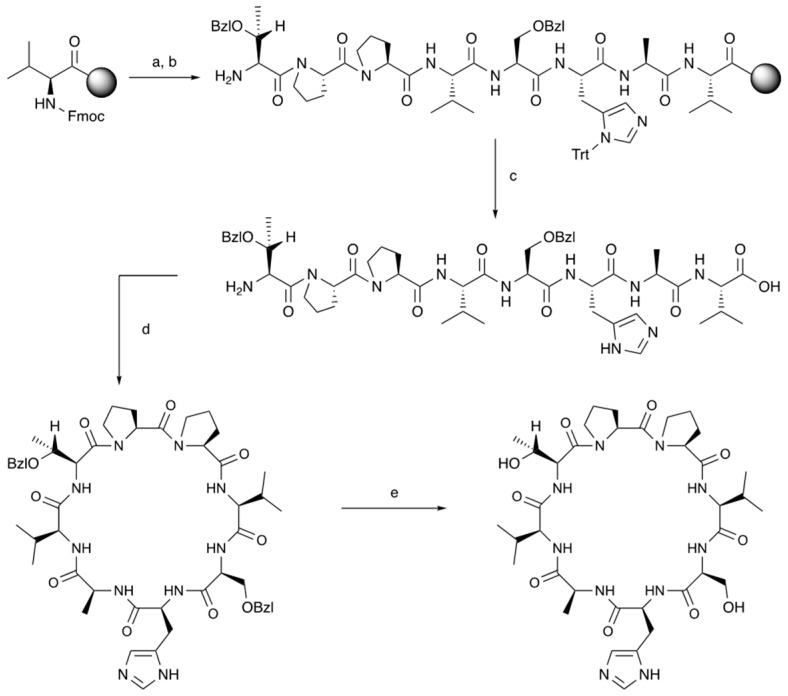
A synthetic scheme to make cyclic ADTHAV peptide. (**a**) Fmoc-deprotection: piperidine/DMF (1:4). (**b**) Coupling reaction in DMF using HCTU, NMM, and Fmoc-amino acids: Fmoc-Ala-OH, Fmoc-Ser(Bzl)-OH, Fmoc-Val-OH, Fmoc-Pro-OH, Fmoc-His(Trt)-OH, Fmoc-Thr(Bzl)-OH, and Fmoc-Pro-OH. (**c**) (i) Fmoc-deprotection reaction and (ii) peptide cleavage from the resin: TFA/H_2_O/TIPS (94:3:3) at room temperature and 2-h reaction time. (**d**) Solution-phase cyclization reaction using HATU/DIEA/peptide (2:4:1) in acetonitrile at room temperature and 24-h reaction time followed by prep-HPLC. (**e**) Final hydrogenation reaction for side-chain deprotection on the peptide was done in a mixture of peptide/H_2_/Pd/C at room temperature and 24-h reaction time.

The goal of this study was to design and synthesize novel cyclic peptides derived from HAV6 and ADTC5 peptides to improve their BBB modulatory activity and selectivity by forming a peptide bond from the N- to C-termini (Table 1, Figure 1). The hypothesis is that the formation of N-to-C-termini cyclic peptides increases the backbone rigidity of the peptide to improve binding affinity and selectivity for the EC1 domain of cadherin as well as improving plasma stability of the cyclic peptide compared to its parent linear peptide. Previously, cHAVc3 and ADTC5 cyclic peptides were formed using a disulfide bond between two Cys residues at the N- and C-termini; these cyclic peptides have enhanced BBB modulatory activity and plasma stability compared to their parent linear peptides [19,20]. However, a disulfide bond in a cyclic peptide can be reduced by glutathione in plasma to form a linear peptide that may have lower biological activity. Thus, we designed stable N-to-C-termini cyclic peptides (i.e., HAVN1 and HAVN2) derived from HAV6 and cyclic ADTHAV peptide utilizing a combination of sequences from ADT and HAV peptides (Table 1, Figure 1). A new cyclization method was successfully developed to make N-to-C-termini cyclic peptides using acetonitrile for easy solvent evaporation rather than using dimethylformamide (DMF), which is difficult to evaporate or remove (Figure 2). The activities of new cyclic peptides were compared to current linear HAV6 and cyclic ADTC5 in delivering IRDye800CW-labeled IgG mAb into the brains of C57BL/6 mice. The effects of the new cyclic peptides on IgG mAb deposition in other organs such as liver, kidney, spleen, lung, and heart were also determined to assess potential off-target tissue effects or side effects of these cadherin peptides.

## 2. Materials and Methods

### 2.1. Chemicals, Reagents, and Animals

The reagents (i.e., trifluoro acetic acid (TFA), hydrogen gas, Pd/C catalyst, triisopropylsilane (TIPS), *O*-(1*H*-6-Chlorobenzotriazole-1-yl)-1,1,3,3-tetramethyluronium hexafluorophosphate (HCTU), 1-[Bis(dimethylamino)methylene]-1*H*-1,2,3-triazolo[4,5-*b*]pyridinium 3-oxide hexafluorophosphate (HATU), *N*-[(Dimethylamino)-1*H*-1,2,3-triazolo-[4,5-*b*]pyridin-1-ylmethylene]-*N*-methylmethanaminium hexa-fluorophosphate *N*-oxide (HATU), diisopropyl ethyl amine (DIEA)) and solvents (i.e., acetonitrile, methanol) were purchased from Sigma Aldrich Chemical Company (St. Louis, MO, USA) and Fisher Scientific Inc. (Hampton, NH, USA). Gyros Protein Technologies Inc. (Tucson, AZ, USA) was the vendor for all Fmoc-protected amino acids for peptide synthesis. IRDye800CW donkey anti-goat IgG was obtained from LI-COR Inc. (Lincoln, NE, USA). All animal studies were carried out under the approved animal protocol (AUS-75-13; Approval Date: 03/26/2019) granted by Institutional Animal Care and Use Committee (IACUC) at The University of Kansas. Animal Care Unit (ACU) personnel and veterinarians were involved in the care of the animals used in this study.

### 2.2. Peptide Synthesis and Purification 

A Tribute solid-phase peptide synthesizer (Gyros Protein Technologies, Inc., Tucson, AZ, USA) with Fmoc chemistry was used to synthesize all linear peptide precursors (Table 1). The HAV6 and linear precursors for cyclic ADTC5 peptide were synthesized using amide resin and were cleaved from the resin with a cocktail mixture of 89% TFA:5% phenol:3% H_2_O:3% TIPS. The linear precursors for N-to-C-termini cyclic peptides (i.e., HAVN1, HAVN2, and ADTHAV) were synthesized using Fmoc-Val-Wang resin (Figure 2). The carboxylic acid and alcohol groups on the side chains were protected with benzyl groups. The peptides were cleaved using a 94% TFA: 3% H_2_O: 3% TIPS cocktail solution. The TFA solutions of linear HAV6, ADTC5, and ADTHAV were added into cold diethyl ether to precipitate the peptide. In contrast, the cleavage solutions of linear HAVN1 and HAVN2 were directly concentrated by rotary evaporator to yield the crude peptides that were further lyophilized.

To form cyclic ADTC5, a very low concentration of linear peptide precursor without any protecting groups was dissolved in bicarbonate buffer solution at pH 9.0; and then, the solution was then bubbled with air to oxidize the two thiol groups in the Cys residues to form a disulfide bond. The end result produced cyclic ADTC5 peptide in a monomeric form with low side products as dimers, trimers, and oligomers. The desired monomer was purified by semi-preparative HPLC using a C18 column Waters XBridge C18 (19 mm × 250 mm, 5 μm particle size; Waters Corporation, Milford, MA, USA). The mobile phase consisted of solvents (A) H_2_O: ACN: TFA (94.9:5:0.1) and (B) acetonitrile with a gradient of 40% B (0 min), 40–100% B (17 min), 100% B (2 min), 100–40% B (2 min), and 40% B (6 min). Before combining the collected fractions, each fraction was evaluated by analytical HPLC using a C18 column (Luna C18, 4.6 mm × 250 mm, 5 μm particle size, 100 Å; Phenomenex, Inc., Torrance, CA, USA) to check for purity, and the pure fractions were pooled, concentrated, and lyophilized. 

**Figure 3:**
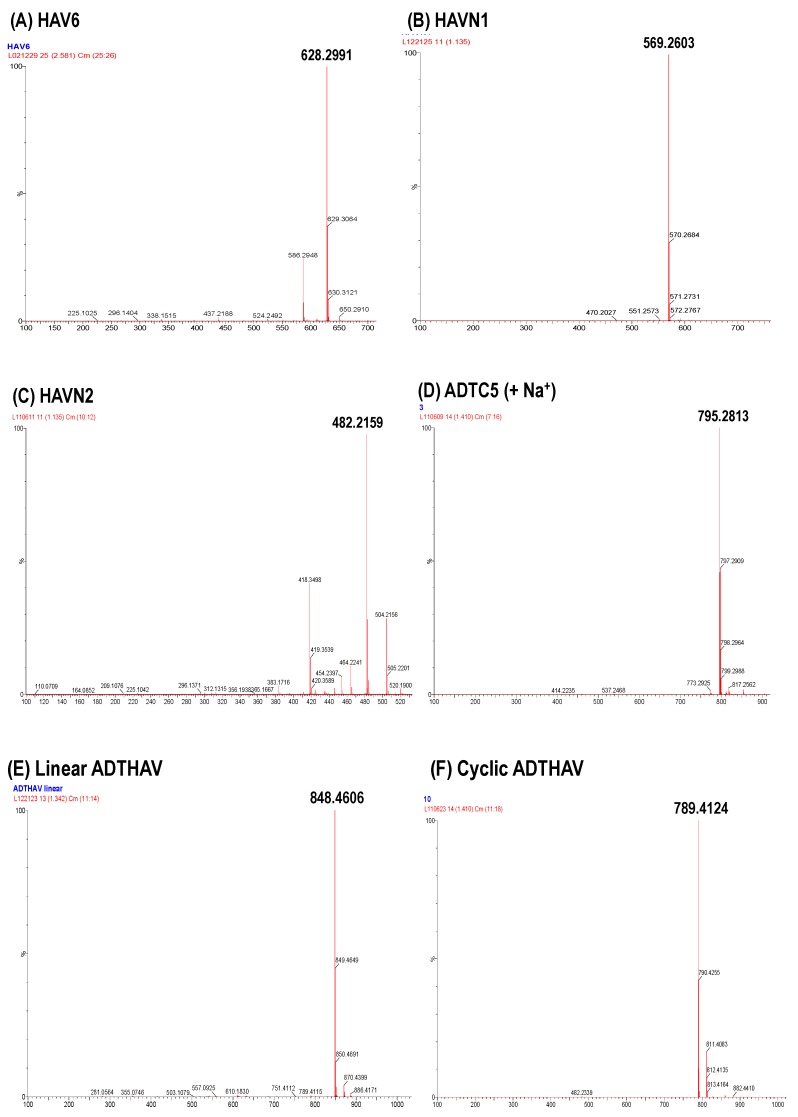
Mass spectra of (**A**) HAV6, (**B**) HAVN1, (**C**) HAVN2, (**D**) ADTC5, (**E**) linear ADTHAV, and (**F**) cyclic ADTHAV peptides to identify the synthesized molecules.2.3. In Vivo Delivery of IRdye800CW IgG mAb.

The N-to-C-termini cyclizations to produce cyclic ADTHAV, HAVN1, and HAVN2 were carried out in solution phase (Figure 2). In this case, the acid and alcohol functional groups on the side chains of the peptide were protected with benzyl ester and ether groups that were removed after cyclization. The optimized molar ratio of peptide: HATU:DIEA for the cyclization reaction was 1:2:4, and the cyclization reaction was done in dilute solution (~6.0 mM peptide) in acetonitrile (ACN). In this case, three separate solutions were prepared: (1) 6.3 mmol peptide in 50 mL of acetonitrile, (2) 12.6 mmol HATU in 50 mL acetonitrile, and (3) 25.2 mmol DIEA in 1 L of acetonitrile. The solutions of peptide and HATU were both added slowly from two different peristaltic pumps into the DIEA solution over 4 h, and the mixture was stirred overnight. The completion time for the cyclization reaction was monitored using mass spectrometry every 4 h to observe the disappearance of the linear precursor and the appearance of the cyclic peptide. After confirming the complete formation of the cyclic peptide, the acetonitrile was removed by rotary evaporator. A C18 semi-preparative HPLC column was used to isolate the cyclic peptide, and the pure peptide was lyophilized. The cyclic peptide was dissolved in methanol and subjected to hydrogenation reaction under balloon pressure in the presence of Pd/C catalyst overnight to remove benzyl ester and ether protecting groups. The final product was purified by semi-preparative HPLC, and the identity of the cyclic peptide was confirmed using mass spectrometry (Figure 3).

The activity of each peptide in enhancing BBB penetration was evaluated by delivering IRdye800CW donkey anti-goat IgG mAb in C57BL/6 mice; the amounts of mAb in the brain were determined using near infrared fluorescence (NIRF) imaging. Each group contains 3 mice per group (*n* = 3) with a mixture of male and female mice, selected randomly for each arm of the study. The injection solution was prepared by adding 600 µL PBS into 0.5 mg lyophilized IgG mAb; then, approximately 1.5 mg lyophilized peptide was added into the mixture yielding the injectable formulation. A 100 µL solution of a mixture containing IgG mAb (21.6 nmol/kg) along with 13 µmol/kg peptide was administered via tail vein. As a control, 100 µL of IgG mAb alone was administered via i.v. route. After the delivered molecules had been circulating for 15 min, the mice were sacrificed; then, a mixture of PBS with 0.5% Tween-20 was administered for cardiac perfusion to remove the blood and delivered molecules from the brain microvessels. The brain and other organs such as lung, heart, spleen, liver, and kidney were harvested and rinsed with PBS. The isolated organs were scanned with Odyssey^®^ CLx for mAb quantification.

The brain deposition of IgG mAb was also quantified by NIRF imaging in brain homogenates. The isolated brains were mechanically homogenized in 2.0 mL of PBS. To make the standard solutions, IRDye800CW IgG mAb stock solution (70 µg/mL) was prepared; it was then diluted with various amounts of PBS to make six different mAb concentrations. To generate a calibration curve, the brain homogenate (200 µL) was aliquoted out to a 96-well plate. A total of 10 µL of each concentration of IgG mAb was added to three different wells of blank brain homogenates. The standard spiked homogenates were at a range of 10–200 ng/mL IgG mAb in brain homogenate. The wells were scanned using the Odyssey^®^ CLx scanner, and the signal intensities vs. concentrations of mAb per gram of brain were used to generate a calibration curve.

### 2.3. Statistical Analysis

ANOVA with Student–Newman–Keuls was used to compare the data for determining statistical significance for IgG mAb deposition in the brains. A *p*-value of less than 0.05 was used as a criterion for a significant difference in data comparison. Numeric results are presented as: mean ± SEM.

## 3. Results

### 3.1. Peptide Synthesis and Purification

The solid-phase peptide synthesizer was used to generate all peptides used in this study, and the synthesis was done using Fmoc amino acids. A high yield of crude peptide was normally found for peptide synthesis. The cyclization reaction to make cyclic ADTC5 peptide was uneventful and generated a mostly monomeric cyclic peptide. The syntheses of N-to-C-termini cyclic peptides were done by a combination of solid-phase and solution-phase synthetic methods (Figure 2). As an example, a linear precursor ADTHAV peptide with uncapped N- and C-termini (TPPVSHAV-OH) was synthesized using solid-phase peptide synthesis, and the side chains of the Asp, Ser, and Thr residues were protected with benzyl ester and ether protecting groups. These protecting groups were maintained during the cleavage reaction to remove the peptide from the resin. After semi-preparative HPLC purification, the isolated product was considered acceptable when it had >96% purity, as determined by analytical HPLC with a C18 column. The cyclization was done in acetonitrile in high dilution to favor intramolecular peptide bond formation and to prevent formation of dimers, trimers, and oligomers. The major product was a monomeric cyclic peptide, and the exact mass of each N-to-C-termini cyclic peptide is shown in Table 1 and Figure 3, where the molecular mass of the cyclic peptide was 18 amu (−H_2_O) less than the precursor linear peptide.

### 3.2. In Vivo Delivery of IRdye800CW IgG mAb

The new cyclic peptides were compared to ADTC5 and HAV6 peptides by evaluating their activities in delivering IgG mAb into the brains of C57BL/6 mice. As a negative control, IgG mAb was delivered in PBS without peptide. Previously, ADTC5 has been shown to improve brain delivery of IgG mAb, which can serve as a positive control. Cyclic HAV peptides (i.e., HAVN1, HAVN2) and linear HAV6 were evaluated to test whether the formation of cyclic peptides could improve their BBB modulatory activity. Cyclic ADTHAV peptide was formed via a combination of ADTC5 and HAV6 sequences to test the potential additive activity of the two sequences. Because ADTC5 and HAV6 bind to two different binding sites on the EC1 domain, it is proposed that the activity of cyclic ADTHAV is also due to its binding to two different binding sites on the EC1 domain.

**Figure 4:**
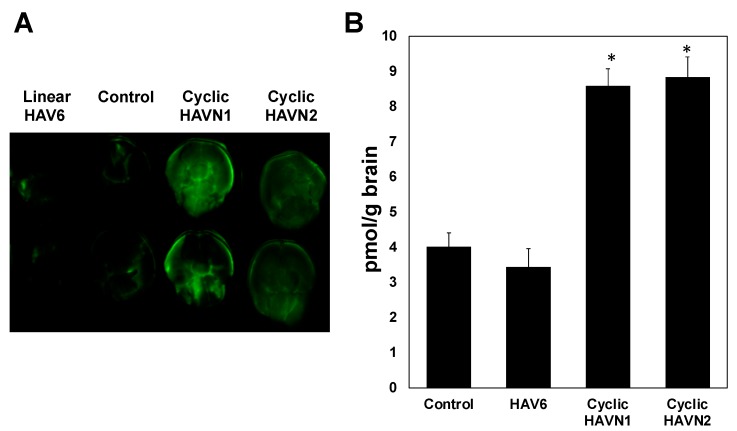
(**A**) The levels of brain deposition of IRdye800CW-IgG mAb were qualitatively shown by NIRF imaging after administration of IgG mAb (21.6 nmol/kg) alone as a control or along with linear HAV6, cyclic HAVN1, or cyclic HAVN2 (13 µmol/kg) in C57BL/6 mice. (**B**) IRdye800CW-IgG mAb brain deposition was determined quantitatively using NIRF imaging in pmol/g brain after delivery of IgG mAb alone (21.6 nmol/kg) or delivered with HAV6, HAVN1, or HAVN2 (13 µmol/kg) in C57BL/6 mice. The asterisk (*) designates a significant difference in HAVN1- or HAVN2-treated groups compared to control with *p* < 0.05. Error bars show the mean ± SEM with the number of animals, *n* = 3, for each group.

**Figure 5:**
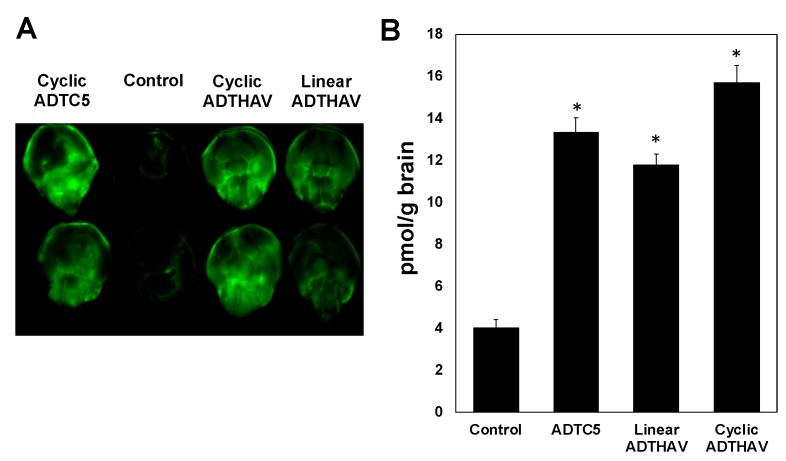
(**A**) The qualitative brain deposition of IRdye800CW-IgG mAb as NIRF images after its i.v. administration alone (21.6 nmol/kg) as a control or together with cyclic ADTC5, linear ADTHAV, or cyclic ADTHAV (13 µmol/kg) in C57BL/6 mice. (**B**) Quantitative determination of IRdye800CW-IgG mAb brain deposition in pmol/g brain after its administration (21.6 nmol/kg) without peptide as a control group or in the presence of ADTC5, linear ADTHAV, or cyclic ADTHAV (13 µmol/kg) in C57BL/6 mice. The asterisk (*) indicates a significant difference in cyclic ADTC5-, linear ADTHAV-, or cyclic ADTHAV-treated groups compared to control with *p* < 0.05. Error bars show the mean ± SEM with the number of animals, *n* = 3, for each group.

A calibration curve was generated to determine the amount of IgG mAb in the brain by spiking blank brain homogenates with a concentration range from 10 to 200 ng/mL, and good linearity with *R*^2^ ≥ 0.98 was achieved. The results showed that HAV6 did not enhance brain delivery of IgG mAb compared to the control, i.e., IgG mAb alone (Figure 4, *p* > 0.05) while IgG mAb brain delivery was significantly enhanced by cyclic HAVN1 and HAVN2 peptides compared to HAV6 and control (Figure 4). These results indicate that cyclic peptide formation increases BBB modulatory activity of HAV peptide. The average amounts of IgG mAb in the brains of HAV6-treated and control animals were 3.4 ± 0.4 and 4.0 ± 0.5 pmol/g brain, respectively. In contrast, the average amounts of mAb in the brains of cyclic HAVN1- and HAVN2-treated mice were 8.6 ± 0.5 and 8.8 ± 0.6 pmol/g brain, respectively. The BBB modulatory activities of ADTC5, linear ADTHAV, and cyclic ADTHAV were also compared to the control (Figure 5). The brain delivery of IgG mAb by linear ADTHAV, cyclic ADTHAV, and ADTC5 was significantly better than in the PBS control. The average brain deposition of IgG mAb were 11.8 ± 0.5, 15.7 ± 0.8, and 13.3 ± 0.7 pmol/g brain for linear ADTHAV, cyclic ADTHAV, and ADTC5, respectively.

**Figure 6:**
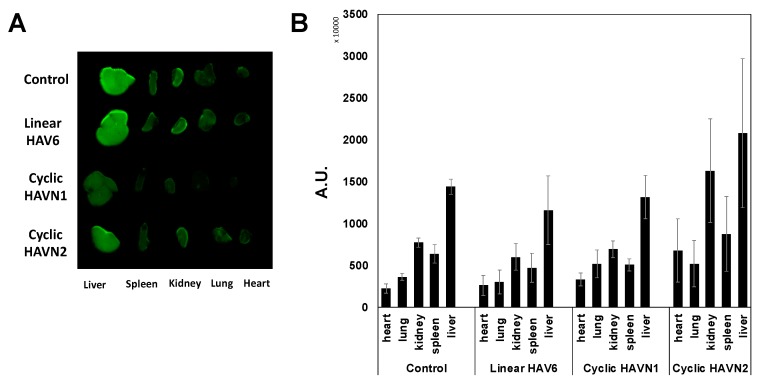
The effects of linear HAV6, cyclic HAVN1, and cyclic HAVN2 peptides on the peripheral organ deposition of the IRdye800CW-IgG mAb in heart, lung, kidney, spleen, and liver determined using NIRF signal intensity (**A**) qualitatively and (**B**) quantitatively in absorption units (A.U.). The IgG mAb deposition was measured by the total NIRF image intensity in each organ. There is no significant difference in the IgG mAb signal intensities for each organ when comparing the control group and peptide-treated group with *p* > 0.05. Error bars show the mean ± SEM with the number of animals, *n* = 3, for each group.

**Figure 7:**
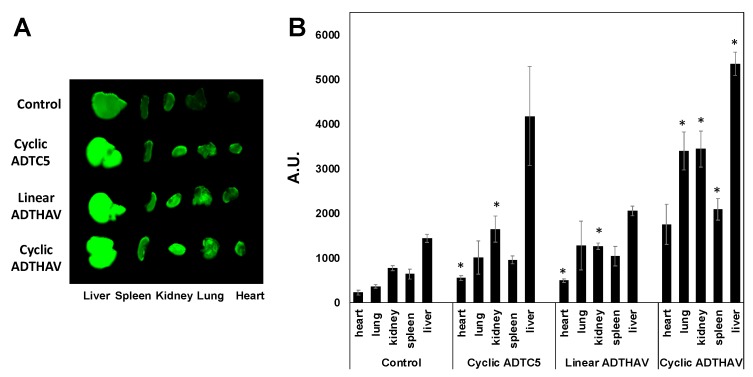
The effects of cyclic ADTC5, linear ADTHAV, and cyclic ADTHAV peptides on the peripheral organ deposition of the IRdye800CW-IgG mAb in heart, lung, kidney, spleen, and liver determined using NIRF signal intensity (**A**) qualitatively and (**B**) quantitatively in absorption units (A.U.). The IgG mAb deposition was measured by the total NIRF image intensity in each organ. There are significance differences in the IgG mAb signal intensities for kidney and heart of ADTC5- or linear ADTHAV-treated mice compared to control (* *p* < 0.05). There are significant differences in the IgG mAb signal in lung, kidney, spleen, and liver from the cyclic ADTHAV-group compared to the control group (* *p* < 0.05). Error bars show the mean ± SEM with the number of animals, *n* = 3, for each group.

The effects of peptides in the deposition of IgG mAb in other organs such as liver, kidney, heart, spleen, and lungs were compared to the control. There was no significant difference in IgG mAb deposition in other organs for HAV6-, HAVN1- and HAVN2-treated animals compared to the control animals (Figure 6; *p* > 0.05). These results suggest that these BBB-modulating peptides do not have a significant impact on other organs. In contrast, ADTC5 and linear ADTHAV peptides have significant effects on the distribution of IgG mAb in the heart and kidney when compared to the control (Figure 7). Moreover, there were significant increases in deposition of IgG mAb in liver, kidney, spleen, and lungs for cyclic ADTHAV peptide when compared to the control (Figure 7; *p* < 0.05).

## 4. Discussion

Treating brain diseases is a challenge as it is difficult to deliver drugs through the BBB into the brain. As many approaches to solve this problem have failed clinically, it is important to investigate new methods to improve brain delivery of molecules for diagnosing and treating brain diseases. This study was focused on designing, synthesizing, and evaluating new cyclic peptides for modulating the BBB to increase paracellular protein delivery into the brain. Our previous studies showed that ADTC5 and HAV6 peptides modulate the paracellular pathways of the BBB and increase the permeation of small marker or drug molecules, peptides, and proteins into the brain [19,20,21,22,23]. In recent studies, the activities of ADTC5 and HAV6 to deliver proteins into the brain were compared in C57BL/6 mice. Both ADTC5 and HAV6 significantly enhanced the brain delivery of 15 kDa lysozyme compared to the control; however, ADTC5 was significantly better than HAV6 in delivering 15 kDa lysozyme into the brain. HAV6 (13 µmol/kg) did not significantly enhance brain delivery of albumin at a low dose (21.6 nmol/kg), while ADTC5 (13 µmol/kg) significantly enhanced brain delivery of albumin compared to the control. In a separate study, when galbumin (600 nmol/kg) was delivered at a 27 times higher dose than NIRF-labeled albumin (21.6 nmol/kg) using HAV6 in living mice, there was a significant brain deposition of galbumin found as detected by magnetic resonance imaging (MRI) [22]. This indicates that the dose of delivered protein can influence the amount of protein deposition in the brain. It is proposed that HAV6 created a small subpopulation of large pores in the intercellular junctions that allow for sufficient amounts of galbumin, when delivered at a high dose, to enter the brain to be detected by magnetic resonance imaging (MRI). As shown here, HAV6 could not enhance the delivery of IgG mAb, while the ADTC5 could. From this, it was clear that ADTC5 is a better BBB modulator than HAV6 for delivering proteins into the brain. The difference in activity of HAV6 and ADTC5 peptide is due to their binding properties to the EC1 domain of cadherin.

Cadherin peptides interrupt the cadherin–cadherin interactions by binding to the extracellular domains of cadherins allowing delivered molecules to pass through the intracellular junctions of the BBB. HSQC NMR and molecular modeling experiments showed that ADT and HAV peptides bind to different sites on the EC1 domain of E-cadherin [26]. Using the X-ray structure of C-cadherin as a model, it is seen that the cadherin molecules can form *cis*- and *trans*-interactions with each other in a homotypic fashion [27]. The *cis*-cadherin interactions were formed via binding of the EC1 domain from one cadherin molecule to the EC2 domain of another cadherin, both of which protrude from the same cell membranes. The *trans*-cadherin interactions were formed by binding between the EC1 domain of a cadherin molecule from one cell membrane to the EC1 domain of another cadherin molecule from an opposing cell membrane. In this case, a Trp residue at the N-terminus of the first EC1 binds to a hydrophobic pocket in the second EC1 from the opposing cell membranes while another Trp on the second EC1 binds to a hydrophobic pocket at the first EC1. This process is called “domain swapping”. Our NMR studies showed that ADTC5 and its derivatives bind to the hydrophobic pocket of the EC1 in which the “domain swapping” can occur between the two *trans*-cadherin interactions; therefore, ADT peptides inhibit *trans*-cadherin interactions. In contrast, NMR studies also indicated that HAV peptides (i.e., HAV6 and cHAVc3) bind to the EC1 domain of E-cadherin at the interface of *cis*-cadherin interactions; thus, it is proposed that HAV peptides block *cis*-cadherin interactions to open the paracellular pathways [26]. Because cyclic ADTHAV is derived from ADTC5 and HAV6, it is proposed that the activity of cyclic ADTHAV is due to its ability to bind both ADT- and HAV-peptide binding sites to inhibit both *cis*- and *trans*-cadherin.

To improve BBB modulatory activity, the aim of this study was to design and synthesize more potent and selective cyclic peptides derived from HAV6 and ADTC5 sequences and evaluate their BBB modulatory effects in delivering IgG mAb to the brain. Previously, the formation of cyclic peptides has been shown to improve biological activity and plasma stability of peptides. For these reasons, cyclic peptides have been developed successfully as therapeutics in the clinic, and there are more than 60 approved peptide-derived drugs available in the United States and other major markets. Among those, a vast majority are derived from natural products such as antimicrobials or human peptide hormones, including widely applied cyclic peptides such as oxytocin, octreotide, vasopressin, vancomycin, daptomycin, and polymyxinB [28]. Peptide cyclization improves conformational stability and binding properties of peptides to their respective target receptor(s). Various covalent bonds have been utilized to form cyclic peptides by linking two ends of the peptide using a disulfide, amide, lactone, ether, or thioether bond. Peptide cyclization can be formed via backbone-to-backbone (or N-to-C termini), backbone-to-side chain, or side chain-to-side chain. Using a disulfide bond via side chain-to-side chain cyclization is the easiest way to make a cyclic peptide by oxidation of the two thiol groups of Cys residues at the two ends of a peptide. However, the disulfide bond can be reduced in the blood by reducing agents (i.e., glutathione). Nonetheless, many cyclic peptide drugs (e.g., oxytocin, integrilin) are formed with a disulfide bond. The second most popular cyclization method is head-to-tail cyclization via N-to-C (or backbone-to-backbone) peptide bond formation. In nature, many biologically active peptides are N-to-C cyclic peptides such as cyclosporin and bacitracin. Compared to their linear counterparts, cyclic peptides are less flexible or more conformationally rigid, which often enhances their receptor selectivity and biological activities. This rigidity reduces the number of available conformations and decreases the entropy term of the Gibbs free energy. Moreover, N-to-C cyclic peptides have been shown to have higher plasma stability than the parent linear peptides; this is due to the flexibility of linear peptides to readily bind to the active side of exo- and endo-peptidases [29].

In this study, cyclic HAVN1 and HAVN2 peptides enhanced in vivo brain delivery of 150 kDa IgG mAb while the parent linear HAV6 (Ac-SHAVSS-NH_2_) did not (Figure 4). Similarly, when HAV6 was used to deliver IRdye800CW-albumin at the same dose as IgG mAb, no significant enhancement of albumin brain deposition was observed compared to albumin alone as a control. It was clear that there was an improvement in the BBB modulatory activity of cyclic HAVN1 and HAVN2 compared to that of linear HAV6. It is proposed that the rigidity imposed on the peptide backbone of the cyclic peptide reduced the ensemble of peptide conformations and, as a result, the backbone rigidity improved peptide binding to the target cadherin protein for modulation of the BBB. HAVN1 is a cyclo-hexapeptide with a sequence similar to that of HAV6, while the HAVN2 is a cyclo-pentapeptide with one Ser residue deleted from the sequence of HAV6. It is interesting that the removal of one serine residue did not influence the BBB modulatory activity of HAVN2 compared to HAVN1 in delivering IgG mAb using the presented conditions. In the future, the effects of peptide dose and residence time in the systemic circulation of peptide along with IgG mAb will be evaluated to possibly differentiate the properties of these peptides. Currently, binding affinities of HAVN1, HAVN2, and HAV6 to the EC1 of E- and VE-cadherins are being evaluated using surface plasmon resonance (SPR) and NMR to differentiate these two peptides.

Previously, cyclic cHAVc1 (cyclo(1,8)Ac-CSHAVASC-NH_2_) and cHAVc3 (cyclo(1,6)Ac-CSHAVC-NH_2_) were prepared and compared to a linear HAV4 (Ac-SHAVAS-NH_2_) in modulating the intercellular junctions in vitro and in vivo [19]. In Madin–Darby Canine Kidney (MDCK) cell monolayers, the results showed that cyclic cHAVc1 (cyclo(1,8)Ac-CSHAVASC-NH_2_) has the same junction modulatory activity as HAV4 peptide [19]. However, deletions of the Ala6 and Ser7 residues from cHAVc1 to produce cHAVc3 (cyclo(1,6)Ac-CSHAVC-NH_2_) made cHAVc3 peptide more active compared to HAV4 and cHAVc1 in modulating the intercellular junctions in the cell culture monolayers. Cyclic cHAVc3 peptide was better than a linear HAV4 in improving in vivo brain delivery of gadopentetic acid (Gd-DTPA) as a magnetic resonance imaging (MRI) agent in mice as well as in lowering the TEER values of MDCK cell monolayers [19]. One of the potential reasons for lower activity of cHAVc1 compared to that of cHAVc3 is that cHAVc1 has more backbone flexibility than the smaller cyclic cHAVc3, confirming that backbone rigidity has a strong influence on the cadherin peptide binding selectivity for target cadherin protein.

This study was also aimed at developing cyclic ADTHAV peptide by combining important residues from the ADT peptide (i.e., ADTC5) and HAV peptide (i.e., HAV6). Because ADTC5 and HAV6 bind at different sites on the EC1 domain, combining the two active sequences could provide synergistic activity form both parent peptides on cyclic ADTHAV. In this case, cyclic ADTHAV contains the Thr-Pro-Pro-Val sequence from ADTC5 and Ser-His-Ala-Val sequence from HAV6. The hypothesis is that the combined sequence enhances BBB modulatory activity better than the parent ADTC5 and HAV6. The selection of the Thr-Pro-Pro-Val sequence to build cyclic ADTHAV was due to the importance of this sequence in previous studies in MDCK cell monolayers as well as in vivo BBB in animal models [20,30]. ADT peptides containing the Ala-Asp-Thr-Pro-Pro-Val (ADTPPV) sequence were derived from a bulge region in the EC1 domain of E-cadherin [30]. In NMR [31] and X-ray [32] structures of the EC1 domain of E-cadherin, the Thr-Pro-Pro-Val sequence was also found on the active surface of the EC1 domain. Bocsik et al. showed that the ADT6 peptide (Ac-ADTPPV-NH_2_) reversibly disrupts the intercellular junctions of primary brain endothelial cell monolayers as measured by *trans*-endothelial electrical resistance (TEER) values [33]. Early studies showed that removal of the Ala1 residue from cyclo(1,8)-Ac-CADTPPVC-NH_2_ (ADTC1) to make cyclo(1,7)-Ac-CDTPPVC-NH_2_ (ADTC5) significantly enhanced the ADTC5 activity compared to that of ADTC1 [20]. In contrast, removal of the Val6 residue from ADTC5 to make cyclo(1,6)Ac-CDTPPC-NH_2_ (ADTC6) lowered the activity of ADTC6 compared to ADTC5, suggesting that the Val residue is an important residue in ADT peptides [20]. Binding studies of ADT peptides to the EC1 domain of E-cadherin using NMR and molecular docking indicated that the Thr-Pro-Pro-Val sequence has an important role in binding interactions.

The selection of the Ser-His-Ala-Val sequence as a building block for the cyclic ADTHAV peptide was rationalized from our previous alanine scanning studies [34], the work on cyclic HAV peptides by Williams et al. [35] and our own work with cHAVc3 (see above) [19]. An alanine scanning study was carried out to evaluate the role of each amino acid in HAV6 peptide, and the biological activity of each alanine mutant peptide was evaluated in lowering the TEER values of MDCK cell monolayers [34]. Mutation of the Ser1 to Ala1 residue reduced the junction modulatory activity of the HAV6 mutant; this finding indicates the potential importance of hydrogen bonding from the hydroxyl group Ser1 to the EC1 domain of E-cadherin [34]. Replacing the Val4 residue with Ala4 in HAV6 dramatically reduced the activity of the mutant; thus, the bulky hydrophobic dimethyl group in Val4 is necessary for its binding to a hydrophobic pocket in the EC1 domain. It is interesting to find that replacing the His2 residue with the Ala2 residue did not cause any change in the activity of the mutant. In HAV4 peptide (Ac-SHAVAS-NH_2_), substitution of Ser5 with Ala5 enhanced modulatory activity of the peptide compared to HAV6. It was proposed that the replacement of a neutral hydrophilic Ser residue with a hydrophobic Ala residue improved peptide binding to cadherin; thus, a non-bulky hydrophobic group (i.e., methyl group) in position 5 is favorable for docking into a small hydrophobic pocket on the EC1 domain of cadherin. Taken together, it was shown that the Ser-His-Ala-Val sequence has an important role in the activity of HAV peptides.

Evaluations of the in vivo activities of ADTC5 and linear and cyclic ADTHAV peptides showed that all three peptides were effective in delivering IgG mAb into the brain compared to IgG mAb alone as a control (Figure 5; *p* < 0.05). Cyclic ADTHAV showed a trend of higher activity in delivering IgG mAb compared to linear ADTHAV (*p* = 0.07), whereas there was no significant difference between cyclic ADTHAV and ADTC5 (*p* = 0.20). It is proposed that a short circulation time (15 min) may not be adequate to differentiate BBB modulatory activities of ADTC5 and ADTHAV peptides. In the future, we will evaluate the effects of dose and longer circulation times to further differentiate the in vivo biological activities of all cyclic peptides that were used in the current study. At the same time, the stability of cyclic ADTHAV and ADTC5 in the systemic circulation can also be compared because it is predicted that the plasma stability of ADTC5 will be lower than that of cyclic ADTHAV; this is due to the instability of the disulfide bridge in ADTC5 that could be reduced by glutathione in the blood.

The BBB modulatory activity of cyclic ADTHAV to deliver IgG mAb was significantly better than that of cyclic HAVN2. However, it was not yet clear whether the difference in activity was due to additive activity by the two sequences (Thr-Pro-Pro-Val and Ser-His-Ala-Val) or domination by only one sequence. As mentioned above, there was a trend that cyclic ADTHAV was slightly better than ADTC5. Because it is difficult to differentiate the activities of cyclic ADTHAV and ADTC5 in vivo, we compared their binding properties to the EC1 domain of E-cadherin using SPR experiments in a separate study. After optimizing the SPR running conditions, the EC1 protein was immobilized on the streptavidin chip via the Streptag I sequence on the N-terminus of the EC1 domain followed by injection of various peptide concentrations. The obtained KD for ADTC5 was 26.8 µM, which is in a range similar to that of the KD (35 µM) determined by NMR upon titration of the ^15^N-labeled EC1-domain of E-cadherin using heteronuclear single quantum coherence spectroscopy (HSQC) experiments. It is interesting to find that cyclic ADTHAV has higher binding affinity (KD = 0.114 µM) than that of ADTC5 (KD = 26.8 µM), indicating that cyclic ADTHAV is more selective than ADTC5. In the future, the mechanism of cyclic ADTHAV binding to the EC1-domains of E-cadherin and VE-cadherin will be evaluated.

E-cadherin is found primarily in epithelial cells while VE-cadherin is found in vascular endothelial cells. It has been suggested previously that VE-cadherin on the BBB has “E-cadherin-like” characteristics. Thus, it will be interesting to find out whether peptides derived from E-cadherin can bind to VE-cadherin and modulate VE-cadherin interactions; this question is being investigated by comparing the binding of HAV and ADT peptides to the EC-1 domain of E- and VE-cadherins. Because cadherin-mediated cell–cell adhesion is also found in different organs (i.e., liver, kidney, heart, spleen, and lung), the effects of cyclic peptides on IgG mAb deposition in other organs were determined to evaluate potential side effects. All peptide-treated groups and the control group showed a significantly higher accumulation of IgG mAb in the liver than in spleen, heart, kidney, and lung because most of the metabolic pathways occur in the liver. When comparing the control group to all groups treated with HAV peptides (i.e., HAV6, HAVN1, and HAVN2), there was no significant difference in deposition of IgG mAb within each specific organ. These results suggest that these peptides did not have any significant effect on the peripheral organs. Thus, HAV peptides may have higher specificity in modulating the BBB than the peripheral organs. In contrast, cyclic ADTC5 and linear ADTHAV showed a significant increase in IgG mAb deposition in heart and kidney, as compared to the control, while the cyclic ADTHAV group had a significant increase in IgG mAb deposition in lungs, spleen, kidney, and liver. The strong effect of cyclic ADTHAV in peripheral organs was presumably due to its strong binding affinity to the EC1 domain of E-cadherins; thus, further studies are needed to evaluate the role of different doses in IgG deposition in peripheral organs.

The duration of time of the BBB opening is an important factor to consider for using cadherin peptides to deliver molecules to the brain. This is because it is dangerous to keep the BBB open for a long period of time, allowing unwanted molecules to enter the brain. However, it is necessary to maintain the opening for an adequate amount of time to allow the delivered molecule to enter the brain with the necessary dose. For example, HAV6 has an opening window of less than 1 h to allow a small molecule such as IRdye-R800 and gadopentetic acid (Gd-DTPA) to enter the brain, while ADTC5 has a duration of opening from 2 to 4 h for brain delivery of Gd-DTPA [20,21]. Using a high dose of 65 kDa galbumin, HAV6 can increase the BBB penetration of galbumin when they are delivered together; however, a 10-min delay between the delivery of HAV6 and galbumin does not allow the galbumin to cross the BBB [22]. Thus, the BBB window of opening by HAV6 for a large protein such as galbumin is short (<10 min). In contrast, the opening created by ADTC5 for galbumin is between 10 and 40 min. It should also be noted that ADTC5 could not deliver 220 kDa fibronectin, indicating that there is a cut-off size of molecules that can be delivered by ADTC5. The results suggest that each peptide produces various populations of pore sizes that can collapse in a time-dependent manner, and each peptide has a cut-off size of molecules that can be delivered through the BBB.

## 5. Conclusions

In this study, we have designed and synthesized novel cyclic peptides that can effectively modulate the BBB to enhance the delivery of mAb into the brains of C57BL/6 mice. The new peptides were designed based on HAV6 and ADTC5 peptides, which modulate cadherin–cadherin interactions in the BBB to improve the delivery of small, medium, and large molecules, including proteins. We have shown that cyclic HAVN1 and HAVN2 peptides have better BBB modulatory effects than their linear counterpart, HAV6. Cyclic ADTHAV with a combination of ADT and HAV sequences has better binding affinity to the EC1 domain of E-cadherin than does ADTC5. Linear and cyclic ADTHAV can significantly enhance brain delivery of IgG mAb compared to the control, and they influence the deposition of IgG mAb in peripheral organs while no HAV peptides influence the deposition of IgG mAb in these organs. In the future, all cyclic peptides will be evaluated for their effects on BBB duration of opening in delivering various size molecules as well as the effects of dose and circulation in the deposition of delivered proteins in the brain. The future direction of this project is to design cadherin peptides that can modulate cadherins in the BBB without affecting cadherins in other organs; thus, the potential side effects of these peptide modulators can be minimized.

## Figures and Tables

**Table 1:** Peptide Names and Peptide Sequences.

Peptide	Sequence	Mass (Da)	Exact Mass (Da)
Cyclic ADTC5	Cyclo(1,7)Ac-CDTPPVC-NH_2_	772	795.2813 (+ Na^+^)
Linear ADTHAV	Ac-TPPVSHAV-NH_2_	847	848.4606
Cyclic ADTHAV	Cyclo(1,8)TPPVSHAV	788	789.4124
Linear HAV6	Ac-SHAVSS-NH_2_	627	628.2991
Cyclic HAVN1	Cyclo(1,6)SHAVSS	568	569.2603
Cyclic HAVN2	Cyclo(1,5)SHAVS	481	482.2159
